# The age threshold of the 8th edition AJCC classification is useful for indicating patients with aggressive papillary thyroid cancer in clinical practice

**DOI:** 10.1186/s12885-020-07636-0

**Published:** 2020-11-30

**Authors:** Krzysztof Kaliszewski, Dorota Diakowska, Łukasz Nowak, Beata Wojtczak, Jerzy Rudnicki

**Affiliations:** 1 Department of General, Minimally Invasive and Endocrine Surgery, Wroclaw Medical University, 50-556 Wroclaw, Borowska Street 213, Wroclaw, Poland; 2 Department of Nervous System Diseases, Faculty of Health Science, Wroclaw Medical University, Wroclaw, Poland; 3 Department of Urology and Urological Oncology, Wroclaw Medical University, Wroclaw, Poland

**Keywords:** Papillary thyroid cancer, Aggressive, Classification, Elderly

## Abstract

**Background:**

Papillary thyroid cancer (PTC) is unique among cancers in that patient age is a consideration in staging. One of the most important modifications in the 8th Edition of the American Joint Committee on Cancer (AJCC) classification is to increase the age cut off for risk stratification in PTC from 45 to 55 years. However, whether this cut off is useful in clinical practice remains controversial. In the present study, we assessed how well this new age threshold stratifies patients with aggressive PTC.

**Methods:**

We retrospectively analyzed the clinicopathological features and overall survival rate of patients with PTC admitted to and surgically treated at a single surgical center. The study protocol was divided into two series. In each series all patients (*n* = 523) were divided in 2 groups according to age cut off. In the first series (cut off 45) patients < 45 (*n* = 193) vs. ≥45 (*n* = 330) were compared, and in the second series (cut off 55) patients < 55 (*n* = 306) vs. ≥55 (*n* = 217) were compared.

**Results:**

The rate of the prevalence of locally advanced disease (pT3 and pT4) was significantly higher in the patients above 55 years old than in those below 55 years old (*p* = 0.013). No significant differences were found for this parameter in series with cut off point 45 years old. A significantly higher risk of locally advanced disease T3 + T4 (OR = 4.87) and presence of LNM (N1) (OR = 3.78) was observed in ≥45 years old group (*p* = 0.021 and *p* < 0.0001, respectively). More expressive results were found for the patients ≥55 years old group, where the risk of locally advanced disease (T3 + T4) was higher (OR = 5.21) and LNM presence was OR = 4.76 (*p* < 0.001 and *p* < 0.0001, respectively). None of the patients below 55 years old showed distant metastasis, but 19 patients above 55 years old showed M1 (p < 0.0001). In older patients group (≥55 years old) we observed deaths related thyroid cancer in 11 individuals.

**Conclusions:**

The age cut off of 55 years old for risk stratification proposed by the 8th Edition of AJCC effectively stratifies PTC patients with a poor prognosis, indicating it is likely to be useful in clinical practice.

## Background

The prevalence of papillary thyroid cancer (PTC) has been increasing steadily in many countries for several decades [1]. In some countries, such as the United States, PTC has the fastest rate of increase among all malignant tumors [2]. Importantly, however, most forms of well-differentiated thyroid cancer (WDTC) have a good prognosis, and the 5-year survival rate of PTC patients is generally above 97% [2–4].

The incidence of PTC has been shown to increase with age [2]. Unlike other malignant tumors, patient age is regarded as an independent risk factor for PTC [5, 6]. PTC also presents a much poorer prognosis in elderly people, although the reason for this finding has not been clearly defined [2, 7–9]. Interestingly, PTC is unique among cancers in that patient age is part of staging [7]. Some authors indicate at the necessity of improving the quality of recommendations on the diagnosis and management of thyroid cancer (TC) [9]. Several TC stratification systems use patient age as a tool for supporting decision-making regarding further therapeutic management. For example, the Mayo Clinic introduced the MACIS (metastases, age, completeness of resection, invasion and size) score for stratifying PTC and follicular thyroid cancer (FTC) [10], and the American Joint Committee on Cancer (AJCC) classification system similarly uses patient age for staging and determining the risk stratification score in WDTC [7]. In these systems, age is one of the most important factors for determining further surgical and adjuvant treatment strategies for patients with PTC. In addition to age, other risk factors, including large tumor size, lymph node metastasis (LNM) and distant metastasis, have also been shown to be risk factors for a poor prognosis in WDTC [11]. Singhal et al. [12] revealed that these clinical characteristics vary among pathological subtypes of PTC.

The current 8th Edition AJCC TNM classification system uses an age of 55 years as a cut off point for risk stratification in tumor staging [13]. By contrast, the previous traditional TNM staging system used an age of 45 years [13] as a cut off for staging, and the change to 55 years prompted a debate regarding how clinically well-founded this age cut off point is for PTC risk stratification, with some authors questioning the utility of using either threshold for risk stratification [14] and others proposing that the age threshold for staging PTC should be further increased [8]. For example, a previous study showed that in PTC, overall survival decreases incrementally with age and that the optimal age threshold for PTC patients is 58.5 years [8]. The same researchers [8] also proposed that in terms of the age threshold for risk stratification, PTC should not be evaluated using the same criteria as those used for FTC, and these conditions should instead be viewed as different forms of WDTC. Other authors have proposed that among older patients, there is no suitable age cut off for decreasing surveillance [14]. Hence, while many authors agree that age is one of the most important factors for risk stratification, there is disagreement regarding what the age cut off should be or whether such a cut off should be applied at all [15].

To address this controversy, we analyzed and compared clinical and histopathological characteristics and disease-free and overall survival rates between PTC patients aged ≥55 years and those aged < 55 years. This age cut off was selected in accordance with the 8th Edition of the AJCC classification [13]. We sought to assess how effectively the new age threshold of the 8th Edition of the AJCC classification stratifies patients with aggressive features of PTC.

## Methods

**Fig. 1:**
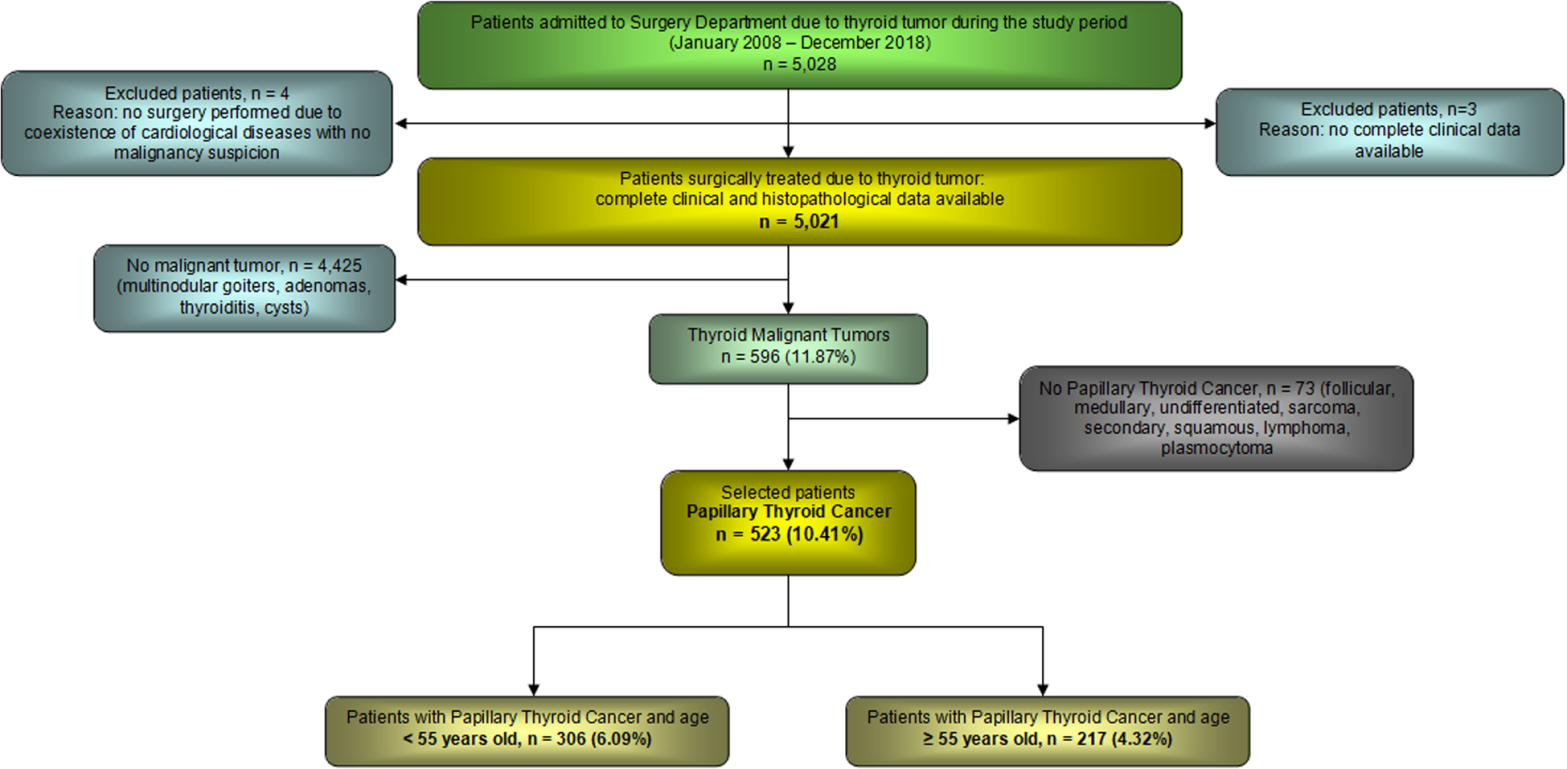
Flowchart of study population. Overview of patient selection from the individuals admitted to thyroid surgery due to thyroid tumor, operated patients, available clinical and histopathological data, and confirmed diagnosis of PTC

We performed retrospective chart reviews of 523 patients admitted to and surgically treated for PTC in the Department of General, Gastroenterological and Endocrine Surgery between 2008 and 2018 (Fig. 1). Diagnostic evaluations and surgical management were performed in accordance with the American Thyroid Association (ATA) guidelines [16]. The inclusion criteria were: the patients with histopathologically confirmed PTC, with complete clinical, pathological and follow up data. All of the included participants underwent ultrasound-guided fine-needle aspiration biopsy (UG-FNAB) within several weeks before surgery. The ultrasound data as microcalcifications, echogenicity, vascularity, and tumor shape were obtained by ultrasonography examination performed by two experienced in thyroid pathology radiologists. Cases of recurrent PTC, with previous head and neck surgery, head and neck radiation exposure, and incomplete clinical or histopathological data were excluded from the study. After surgery, hematoxylin and eosin (H&E)-stained sections were evaluated by two experienced thyroid lesions pathologists to confirm the diagnosis, histopathological features and extent of the malignancy.

Clinical and demographic data were collected on age, sex, UG-FNAB results, type of surgery, and survival time. Pathological characteristics data were collected on tumor size, LNM, distant metastasis, vascular invasion, capsular invasion, microcalcification, multifocality, bilaterality and tumor extension into adjacent tissues (cancer invasion beyond the thyroid capsule). All individuals diagnosed with PTC from both group of patients after operation were routinely sent to the Oncology Center in Gliwice, for consultation and possible adjuvant therapy with radioiodine (RAI).

The study protocol was divided into two series. In each series all patients were divided in 2 groups according to cut off of age at PTC diagnosis (i.e. < 45 vs. ≥45 and <  55 vs. ≥55 years) Then, in each series, patient’s characteristics were compared according to each classification. Obtained results from comparisons within two series were evaluated.

### Statistical analysis

Statistical analyses of the data were performed using Statistica 13.3 software (Tibco Software, Inc., CA, USA). Descriptive data are presented as the number of observations and the percent or as the average ± standard deviation (SD) and 95% confidence interval (95% CI). Qualitative variables were compared using Pearson chi-square and Fisher tests, and quantitative variables were analyzed using Student’s t test for independent samples. Univariate logistic regression was used to analyze the association of age (as the independent variable) with locally advanced disease (pT3 + T4) and lymph node (pN1) or distant (pM1) metastases (as dependent variables). The Kaplan-Meier method and log-rank test were performed to compare the distributions of disease-free survival (DFS) and overall survival (OS) between patients < 45 vs. > 45 years old and <  55vs. > 55 years old. For the purpose of this study we assessed DFS as the length of time after primary surgery during the patients survived without any structural evidence of disease. OS was the length of time in which patients were still alive from the date of PTC surgery. A *p*-value less than 0.05 was considered statistically significant.

## Results

### General demographic and clinical features of PTC patients

The majority of patients (87.2%) were female, and mean age at the time of diagnosis was 50.0 + 12.8 years old. The main patient’s clinical data are presented in the Table 1. There are 433 patients in stage I, 67 patients in stage II, 15 patients in stage III and 8 patients in stage IV according to pTNM classification. Most of the patients (84.9%) were in pT1 tumor stage. In 66.9% patients we did not observe LNM, and in 86% patients distant metastases were not observed.

**Table 1:** Characteristic of PTC patients. Descriptive data were presented as number of observation (percent) or mean + SD

Variable	Total group (n = 523)
Gender:	
Female	456 (87.2)
Male	67 (12.8)
Age at diagnosis (years)	50.00 + 15.38
Age (cutoff 45 years old):	
< 45 years	193 (36.9)
> 45 years	330 (63.1)
Age (cutoff 55 years old):	
< 55 years	306 (58.5)
> 55 years	217 (41.5)
Diagnosis of thyroid malignancy:	
Before surgery	319 (61.0)
After surgery	204 (39.0)
Type of surgery:	
Total	381 (72.8)
Partial	142 (27.2)
Reoperation:	
No	388 (74.2)
Yes	135 (25.8)
pTNM stage:	
I	433 (82.8)
II	67 (12.8)
III	15 (2.9)
IV	8 (1.5)
Tumor stage:	
pT1a	208 (39.8)
pT1b	236 (45.1)
pT2	61 (11.7)
pT3	9 (1.7)
pT4a	3 (0.6)
pT4b	6 (1.1)
Lymph node metastasis:	
pN0	350 (66.9)
pN1a	145 (27.7)
pN1b	6 (1.1)
pNx	22 (4.2)
Distant metastasis:	
pM0	450 (86.0)
pM1	19 (3.6)
pMx	54 (10.4)

### Clinical and histopathological features in PTC patients divided in two groups

In Table 2 we compared demographic, clinical and histopathological features between the two series of both age groups. Significantly higher rates of diagnosis of thyroid malignancy after surgery, advanced pTNM stage, LNM and distant metastasis were observed in the older groups than those < 45 or <  55 years old in both series (for all *p* < 0.05). The rate of the prevalence of locally advanced disease (pT3 and pT4) was significantly higher in the patients above 55 years old than in those below 55 years old (*p* = 0.013). No significant differences were found for this parameter in series with cut off point 45 years old. The study groups did not differ statistically in terms of sex, type of operation or number of reoperations performed.

**Table 2:** Demographic, clinical and histopathological features in PTC patients divided into two age subgroups according cut-off point 45 years old and cut-off point 55 years old. Data are presented as the mean ± SD or number (percent)

Variables	Cut-off point 45 years old			Cut-off point 55 years old		
	PTC patients < 45 years old (n = 193)	PTC patients > 45 years old (n = 330)	p-value	PTC patients < 55 years old (n = 306)	PTC patients > 55 years old (n = 217)	p-value
Age (years)	33.49 + 7.51	59.67 + 9.42	< 0.0001^a^	39.65 + 10.22	64.61 + 7.61	< 0.0001^a^
Sex:			0.460			0.099
Female	171 (88.6)	285 (86.4)		273 (89.2)	183 (84.3)	
Male	22 (11.4)	45 (13.6)		33 (10.8)	34 (15.7)	
Diagnosis of thyroid malignancy:			0.014^a^			0.038^a^
Before surgery	131 (67.9)	188 (57.0)		198 (64.7)	121 (55.8)	
After surgery	62 (32.1)	142 (43.0)		108 (35.3)	96 (44.2)	
Type of surgery:			0.463			0.986
Total	137 (71.0)	244 (73.9)		223 (72.9)	158 (72.8)	
Partial	56 (29.0)	86 (26.1)		83 (27.1)	59 (27.2)	
Reoperation:			0.510			0.687
No	140 (72.5)	248 (75.2)		229 (74.8)	159 (73.3)	
Yes	53 (27.5)	82 (24.8)		77 (25.2)	58 (26.7)	
pTNM stage:			0.004^a^			0.0007^a^
I	172 (89.1)	261 (79.1)		265 (86.6)	168 (77.4)	
II	20 (10.4	47 (14.2)		36 (11.8)	31 (14.3)	
III	1 (0.5)	14 (4.2)		5 (1.6)	10 (4.6)	
IV	0 (0.0)	8 (2.4)		0 (0.0)	8 (3.7)	
Tumor stage (pT):			0.055			0.013^a^
pT1a	72 (37.3)	136 (41.2)		120 (39.2)	88 (40.6)	
pT1b	101 (52.3)	135 (40.9)		150 (49.0)	86 (39,6)	
pT2	18 (9.3)	43 (13.0)		32 (10.5)	29 (13.4)	
pT3	1 (0.5)	8 (2.4)		3 (1.0)	6 (2.7)	
pT4a	0 (0.0)	3 (0.9)		0 (0.0)	3 (1.4)	
pT4b	1 (0.5)	5 (1.5)		1 (0.3)	5 (2.3)	
Lymph node metastasis (pN):			< 0.0001^a^			< 0.0001^a^
pN0	162 (83.9)	188 (57.0)		250 (81.7)	100 (46.1)	
pN1a	26 (13.5)	119 (36.1)		49 (16.0)	96 (44.2)	
pN1b	2 (1.0)	4 (1.2)		3 (1.0)	3 (1.4)	
pNx	3 (1.6)	19 (5.7)		4 (1.3)	18 (8.3)	
Distant metastasis (pM):			0.002^a^			< 0.0001^a^
pM0	178 (92.2)	272 (82.4)		276 (90.2)	174 (80.2)	
pM1	0 (0.0)	19 (5.8)		0 (0.0)	19 (8.8)	
pMx	15 (7.8)	39 (11.8)		30 (9.8)	24 (11.0)	

^a^- statistically significant

*Univariate logistic regression analysis of age ≥ 45 and ≥ 55 years old as risk factor for more advanced PTC entities.* Based on obtained results, we conducted a univariate logistic regression analyses using an age of > 45 years old or > 55 years old as a risk factor for locally advanced tumor. As we shown in Table 3, a significantly higher risk of locally advanced disease T3 + T4 (OR = 4.87) and presence of LNM (N1) (OR = 3.78) was observed in ≥45 years old group (*p* = 0.021 and *p* < 0.0001, respectively). More expressive results were found for the patients ≥55 years old group, where the risk of locally advanced disease (T3 + T4) was higher (OR = 5.21) and LNM presence was OR = 4.76 (*p* < 0.001 and *p* < 0.0001, respectively) (Table 4). None of the patients < 45 and <  55 years old showed distant metastasis, but 19 patients above 55 years old showed distant metastasis (p < 0.0001).

**Table 3:** Univariate logistic regression analysis of age > 45 years old as risk factor for more advanced PTC entities in terms of T, N and M stage. pNx (*n* = 22) and pMx (*n* = 54) were not included in the calculations. Descriptive data are presented as the number (percent), and the results were analyzed by Wald test

*Covariate:* *>* *45 years old*	*pT (n = 523):*		*OR*	*+* *95% CI*	*p-value*
	*pT1 + pT2 (n = 505)*	*pT3 + pT4 (n = 18)*			
< 45 years	191 (37.8)	2 (11.1)	4.87	1.10–21.39	0.021^a^
> 45 years	314 (62.2)	16 (88.9)			
	*pN (n = 501):*		*OR*	*+* *95% CI*	*p-value*
	*pN0 (n = 350)*	*pN1 (n = 151)*			
< 45 years	162 (46.3)	28 (18.5)	3.78	2.38–6.00	< 0.0001^a^
> 45 years	188 (53.7)	123 (81.5)			
	*pM (n = 469):*		*OR*	*+* *95% CI*	*p-value*
	*pM0 (n = 450)*	*pM1 (n = 19)*			
< 45 years	178 (39.6)	0 (0.0)	–	–	< 0.001^a^
> 45 years	272 (60.4)	19 (100.0)			

*PTC* papillary thyroid cancer, ^a^- statistically significant

**Table 4:** Univariate logistic regression analysis of age > 55 years old as risk factor for more advanced PTC entities in terms of T, N and M stage. pNx (n = 22) and pMx (n = 54) were not included in the calculations. Descriptive data are presented as the number (percent), and the results were analyzed by Wald test

*Covariate:* *>* *55 years old*	*pT (n = 523):*		*OR*	*+* *95% CI*	*p-value*
	*pT1 + pT2 (n = 505)*	*pT3 + pT4 (n = 18)*			
< 55 years	302 (59.8)	4 (22.2)	5.21	1.69–16.04	0.001^a^
> 55 years	203 (40.2)	14 (77.8)			
	*pN (n = 501):*		*OR*	*+* *95% CI*	*p-value*
	*pN0 (n = 350)*	*pN1 (n = 151)*			
< 55 years	250 (71.4)	52 (34.4)	4.76	3.16–7.15	< 0.0001^a^
> 55 years	100 (28.6)	99 (65.6)			
	*pM (n = 469):*		*OR*	*+* *95% CI*	*p-value*
	*pM0 (n = 450)*	*pM1 (n = 19)*			
< 55 years	276 (61.3)	0 (0.0)	–	–	< 0.0001^a^
> 55 years	174 (38.7)	19 (100.0)			

*PTC* papillary thyroid cancer, ^a^- statistically significant

### Ultrasound (A) and histopathological (B) features of PTC patients divided into two age subgroups

Ultrasound (A) and histopathological (B) features differed significantly between the older and younger patient groups in two series of test (Table 5). In comparison of both series of selected patients significantly higher rates of:

(A) irregular tumor shape, microcalcifications,
(B) vascular and capsular invasion, extrathyroidal extension, multifocality, bilaterality and multiplicity of foci were observed in ≥45 years old and ≥ 55 years old subjects than in younger counterparts (*p* < 0.05 for all). There were significant differences in:

(A) hypoechogenicity in the series “cut off 45 years old” between younger and older patients (*p* = 0.005), but not in series “cut off 55 years” (*p* = 0.143).

**Table 5:** Ultrasound (A) and histopathological (B) features of PTC patients divided into two age subgroups according cut-off point 45 years old and cut-off point 55 years old. Data are presented as the number of observations (percent)

Variables		Cut-off point 45 years old			Cut-off point 55 years old		
		PTC patients < 45 years old (*n* = 193)	PTC patients > 45 years old (n = 330)	p-value	PTC patients < 55 years old (n = 306)	PTC patients > 55 years old (n = 217)	*p*-value
(A) Ultrasonography	Tumor shape			0.006^a^			0.025^a^
	Regular	109 (56.5)	145 (43.9)		162 (52.9)	93 (42.9)	
	Irregular	84 (43.5)	185 (56.1)		144 (47.1)	124 (57.1)	
	Sharpen margins			0.004^a^			0.009^a^
	Yes	108 (56.0)	142 (42.9)		161 (52.6)	89 (41.0)	
	No	85 (44.0)	188 (57.1)		145 (47.4)	128 (59.0)	
	Microcalcifications:			< 0.0001^a^			< 0.0001^a^
	Yes	67 (34.7)	232 (70.3)		116 (37.9)	183 (84.3)	
	No	126 (65.3)	98 (29.7)		190 (62.1)	34 (15.7)	
	Echogenicity:			0.005^a^			0.143
	Hyperechoic	49 (25.4)	51 (15.5)		65 (21.2)	35 (16.1)	
	Hypoechoic	144 (74.6)	279 (84.6)		241 (78.8)	182 (83.9)	
	Vascularity:			0.002^a^			0.007^a^
	High	82 (42.5)	188 (57.0)		142 (46.4)	127 (58.5)	
	Low	111 (57.5)	142 (43.0)		164 (53.6)	90 (41.5)	
(B) Histopathology	Extrathyroidal extension:			< 0.0001^a^			< 0.0001^a^
	Yes	38 (19.7)	147 (44.6)		66 (21.6)	119 (54.8)	
	No	155 (80.3)	183 (55.5)		240 (78.4)	98 (45.2)	
	Capsular invasion:			< 0.0001^a^			< 0.0001^a^
	Yes	38 (19.7)	147 (44.6)		66 (21.6)	119 (54.8)	
	No	155 (80.3)	183 (55.5)		240 (78.4)	98 (45.2)	
	Vascular invasion			< 0.0001^a^			< 0.0001^a^
	Yes	38 (19.7)	147 (44.6)		66 (21.6)	119 (54.8)	
	No	155 (80.3)	183 (55.5)		240 (78.4)	98 (45.2)	
	Diagnosed as multifocal:			0.002^a^			0.036^a^
	No	156 (80.8)	224 (67.9)		233 (76.1)	147 (67.7)	
	yes	37 (19.2)	106 (32.1)		73 (23.9)	70 (32.3)	
	Diagnosed as bilateral:			0.004^a^			0.002^a^
	No	186 (96.4)	294 (89.1)		291 (95.1)	190 (87.6)	
	Yes	7 (3.6)	36 (10.9)		15 (4.9)	27 (12.4)	
	Number of foci:			0.004^a^			0.0005^a^
	1	169 (87.6)	255 (77.3)		259 (84.6)	165 (76.0)	
	2	22 (11.4)	56 (17.0)		43 (14.1)	35 (16.2)	
	3	2 (1.0)	19 (5.7)		4 (1.3)	17 (7.8)	
	4	0 (0.0)	0 (0.0)		0 (0.0)	0 (0.0)	

^a^- statistically significant

**Fig. 2:**
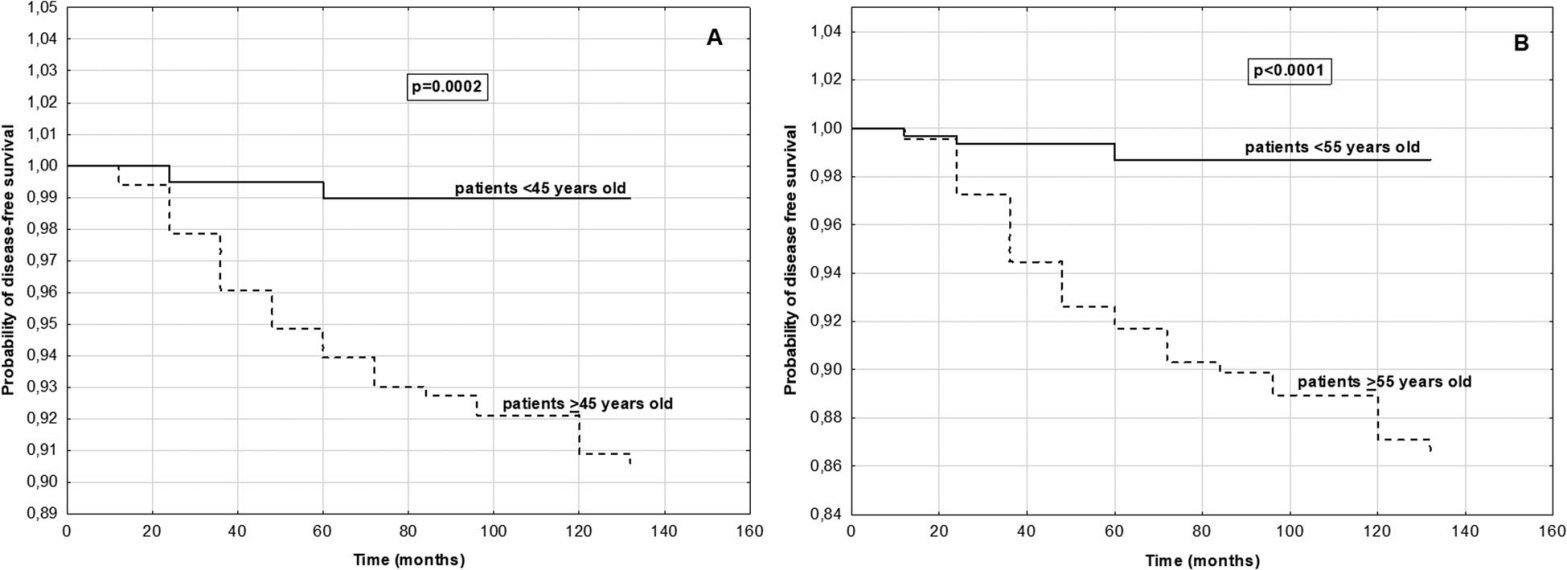
Kaplan-Meier curves comparing probability of disease-free survival of PTC patients with **a**: < 45 years old group and > 45 years old group; **b**: < 55 years old group and > 55 years old group

**Fig. 3:**
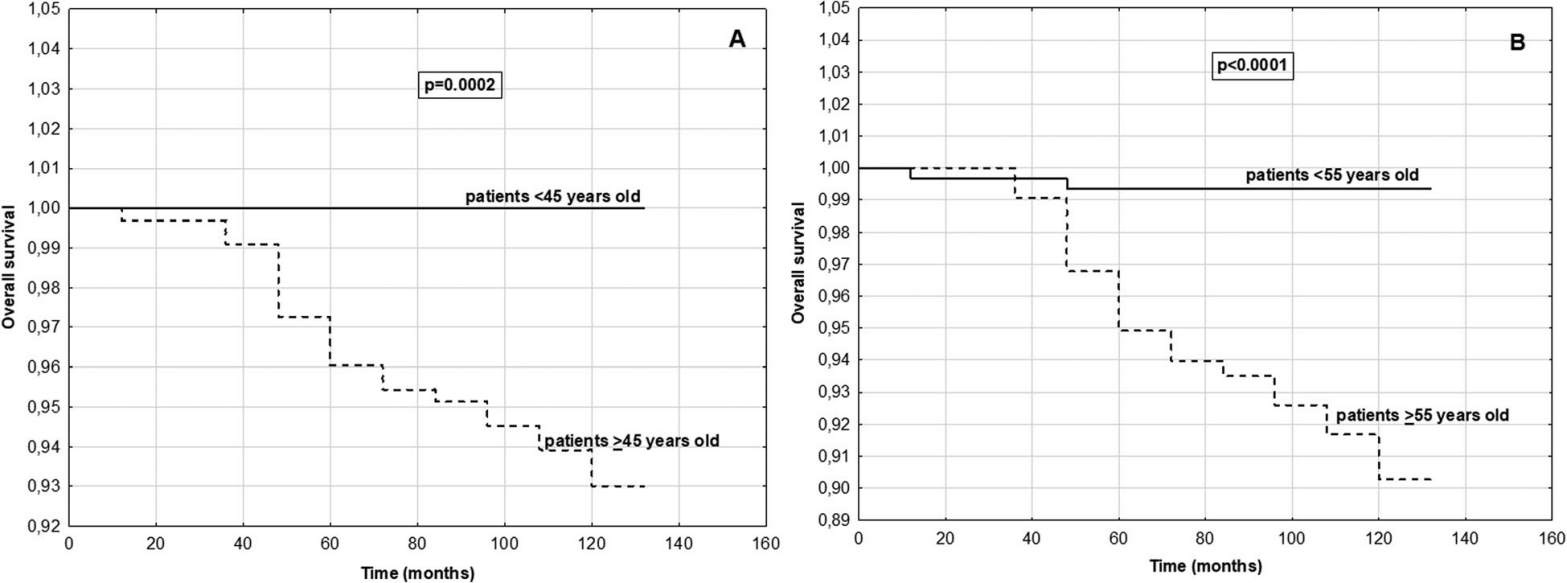
Kaplan-Meier curves comparing probability of survival of PTC patients with **a**: < 45 years old group and > 45 years old group; **b**: < 55 years old group and > 55 years old group

We created models of disease free and overall survival using age cut off point 45 years old and cut off point 55 years old as the predictive variable. The probabilities of disease free survival in PTC patients with cut off 45 years old groups and cut off 55 years old groups during the observation period (2008–2018) are shown in Fig. 2 (Fig. 2). The rate of disease free survival was significantly lower in older patients than in younger patients in two series of test (*p* < 0.05 for both). Our analysis of the ratios of patients who survived over time (indicating overall survival at each time point) in each group from 2008 to 2018 showed that at the final time point, overall survival was significantly lower in older patients than in younger patients in two series of test (p < 0.05 for both) (Fig. 3). In older patients group (≥55 years old) we observed deaths related TC in 11 individuals.

## Discussion

Our findings support our hypothesis, based on the 8th edition of the AJCC classification, that in classical PTC, patients aged ≥55 years have comparatively higher risk of locally advanced disease than those < 55 years. We reveal that the incidence of aggressive pathological features was higher and the incidence of histopathological characteristics associated with an aggressive course much higher in PTC patients in the older group than in those in the younger group. These findings might explain why this age cut off is effective. This may explain our observation that among patients with the same pTNM stage, prognoses were much worse in patients aged ≥55 years than in those aged < 55 years. In our study, we also found that the incidence of pathological features characteristic of aggressive cases of PTC increased with patient age, with individuals with aggressive characteristics of PTC being slightly older than those without such characteristics. To extend the life spans of older patients with PTC, it is crucial to assess all risk factors for aggressive entities of PTC and as well as postsurgical prognosis; while some demographic factors, including sex, are not correlated with aggressive features of PTC, we found that older age is correlated with a more aggressive course.

We observed a higher rate of LNM and distant metastasis in older patients versus younger patient. A significantly higher risk of presence of LNM (N1) was observed in ≥45 years old group, however more expressive results were found for the patients ≥55 years old group, where the risk of locally advanced disease and LNM were higher. None of the patients below 55 years old showed distant metastasis, but 19 patients above 55 years old showed M1. A potential partial explanation for this observation lies in the histopathological characteristics of PTC in these groups of patients. Compared to younger PTC patients, in older PTC patients, we observed a higher number of aggressive features, indicating the potential for more aggressive tumor spread (to distant regions and not only to regional lymph nodes). Consistent with these results, we additionally found that the prevalence of LNM was higher in older than in younger PTC patients.

Our survival analysis further suggested that prognoses are worse in older patients than their younger counterparts, potentially because the incidence of aggressive PTC features is higher in the older group. We noted that forms of PTC without capsular infiltration were much more frequently observed in younger than in older patients, while infiltrative subtypes were more common in older than in younger patients; these findings might explain the better prognoses observed in the younger patients. Similarly, some authors have reported that patients with encapsulated PTC have an excellent prognosis, while those with infiltrative tumors have a comparatively worse prognosis [17, 18]. Importantly, some genetic differences between encapsulated and infiltrative neoplasms have been identified [18]. Nikiforov et al. [17] determined that most patients with encapsulated PTC underwent only lobectomy, and patients treated in this way had a very low risk of adverse outcomes over long-term follow-up. Based on these and similar findings, in 2016, cases of encapsulated PTC were formally classified as noninvasive follicular thyroid neoplasm with papillary-like nuclear features (NIFTP) [17, 19]. Conversely, some authors found that there was no difference in postsurgical outcomes between PTC patients treated with lobectomy vs. thyroidectomy, but also that RAI therapy provided no survival benefit [4, 20–22]. The survival analysis performed in our study suggests that clinicians should consider both patient age and the results of histopathological examinations when treating PTC patients. This approach may save some patients, especially younger individuals with comorbidities, from undergoing unnecessary aggressive therapies. Even the 2015 ATA Management Guidelines for Patients with Differentiated Thyroid Cancer recommends hemithyroidectomy without RAI therapy in low-risk patients [16], and some authors have, based on these guidelines, begun to identify which groups of patients may be at risk of overtreatment with RAI ablation after surgery for low-risk PTC [23].

The situation is completely different in PTMC patients. Ito et al. [24], for example, revealed that there are significant age-specific differences in cancer biology among indolent, clinically silent PTMC and advanced PTC – in particular, the incidences of larger tumors, LNM and disease progression were estimated to be lower in older patients and higher in younger patients. Similar observations were presented by Kim et al. [6], who showed that extrathyroidal extension and LNM were significantly less frequent in older patients with PTMC than in patients with larger PTC. They suggested, based on this finding, that older patients with clinically silent PTMC should be considered for active observation rather than surgical treatment [6]. In our study, we identified some clinical and histopathological characteristics of PTC that, when absent, may permit clinicians to avoid aggressive therapy in older patients. Additionally, we confirmed that among PTC patients one ultrasound feature i.e. microcalcifications and some histopathological features like extrathyroidal extension, capsular and vascular invasion, LNM and distant metastasis are more common in older than in younger patients, and are risk factors for a poor prognosis. We observed these features mainly in patients ≥55 years old. According to some authors, the use of LNM in risk stratification remains controversial [25], while many other studies have presented LNM as a significant risk factor for poor outcomes in PTC [13, 26]. In contrast, other authors have reported inconsistent conclusions concerning the impact of LNM on PTC [25]. Analyses have also shown that in FTC but not PTC, an older age at diagnosis is a significant risk factor for disease-specific mortality [27]. Zhang et al. [28] compared younger, middle-aged and older patients with PTC and found that bilateral LNM was more likely to occur in the older patients (45–65 years old) than in the younger groups. They additionally concluded that the tumors in the older group were more likely than those in the middle and younger groups to show capsular and extrathyroidal invasion. Kim et al. [6] proposed that tumor size and LNM are independent predictors of recurrence in older patients with PTC. De Castro et al. [29] added that tumor size, local extent (T stage) and nodal status (N stage) are also important prognostic factors in patients ≥45 years old. In our study, we observed that some risk factors, such as LNM, capsular invasion and distant metastasis, were more common in older patients than in younger patients. We estimated that age cut off of 55 appears more relevant than 45 to discriminate patients according to N+ and M+. We found that an age of 55 years is a clearly useful cut off threshold for stratifying PTC patients, with patients younger and older than this age having better and poorer prognoses, respectively, consistent with a recent study [14]. Those authors used Cox proportional hazards analysis to assess the association between specific risk factors and the prognosis of WDTC and showed, consistent with our findings, that an age of 55 years is an effective cut-off threshold for risk stratification in PTC patients. This age threshold effectively stratifies patients with aggressive tumors, and our data indicate there is no need to increase this threshold. We confirmed the results reported by Gillanders et al. [30], which supported increasing the age threshold from 45 to 55 years. Because the evidence supporting the use of an age of 45 years as a cut-off for stratification had become controversial, we performed our study and show that an age of 55 years seems very reasonable, contrary to some authors who proposed that no age cut-off is appropriate for significant risk stratification [31, 32]. Our data indicate that an age cutoff of 55 years could help clinicians perform risk stratification. For example, patients aged ≥55 years are more likely than their younger counterparts to present PTC with clinicopathological features pathognomonic for aggressive entities, while < 55 years old very often have indolent, clinically silent PTC. The aggressive clinicopathological features observed in older PTC are likely responsible for their comparatively poorer survival and prognosis. Access to these data may help clinicians deciding whether to perform more radical treatment. Equally important is that clinicians may be able to prevent unnecessary surgery and aggressive postsurgical RAI therapy in older patients with PTC without invasive features on histopathological examination.

Our study has some limitations. First, it is limited by its retrospective design, which prevented adjustment for some confounding factors. Second, this was a single-center analysis; to better understand this issue, multicenter analysis will be necessary. Third, due to the indolence of PTC and PTMC, our study was conducted over a relatively short follow-up period; a longer follow-up period is needed to completely assess the impact of PTC characteristics on prognosis and mortality. All individuals after operation were routinely sent to the oncology center for consultation and potential adjuvant radioiodine treatment (RAI). Due to lack of absolutely all data regarding RAI therapy we did not present these details.

## Conclusions

Overall, our results support the notion that the individual risk stratification and treatment of older patients with PTC is reasonable. PTC is more aggressive in patients aged ≥55 years than in their younger counterparts. This age therefore effectively stratifies PTC patients with a poor prognosis, indicating it is likely to be useful in clinical practice.

## Data Availability

The datasets used and/or analyzed during the current study are available from the corresponding author on reasonable request.

## Abbreviations

PTC: Papillary Thyroid Cancer
AJCC: American Joint Committee on Cancer
LNM: Lymph Node Metastasis
TNM: Tumor Node Metastasis
WDTC: Well-Differentiated Thyroid Cancer
FTC: Follicular Thyroid Cancer
H&E: Hematoxylin and Eosin
PTMC: Papillary Thyroid Microcarcinoma
ATA: American Thyroid Association
UG-FNAB: Ultrasound-Guided Fine-Needle Aspiration Biopsy
SD: Standard Deviation
CI: Confidence Interval
NIFTP: Noninvasive Follicular Thyroid Neoplasm with Papillary-like Nuclear Features
RAI: Radioactive Iodine
